# Out of the Shadow: Blue Light Exposure Induces Apoptosis in Müller Cells

**DOI:** 10.3390/ijms232314540

**Published:** 2022-11-22

**Authors:** Agnes Fietz, José Hurst, Sven Schnichels

**Affiliations:** Center for Ophthalmology Tübingen, University Eye Hospital Tübingen, 72076 Tübingen, Germany

**Keywords:** ROS, oxidative stress, Müller cells, blue light, neurodegeneration, apoptosis, cell death, red light, p53, AMD

## Abstract

Awareness toward the risks of blue light (BL) exposure is rising due to increased use of BL-enriched LEDs in displays. Short-wave BL (400–500 nm) has a high photochemical energy, leading to the enhanced production of reactive oxygen species (ROS). BL potentially plays a role in causing dry eye, cataracts, and age-related macular degeneration (AMD). The effect of BL on retinal pigment epithelium cells (RPEs) or photoreceptors has been extensively investigated. In contrast, only a few studies have investigated the effects of BL exposure on Müller cells (MCs). This is mainly due to their lack of photosensitive elements and the common assumption that their reaction to stress is only secondary in disease development. However, MCs perform important supportive, secretory, and immune functions in the retina, making them essential for retinal survival. Increased oxidative stress is a key player in many retinal diseases such as AMD or glaucoma. We hypothesize that increased oxidative stress can also affect MCs. Thus, we simulated oxidative stress levels by exposing primary porcine MCs and human MIO-M1 cells to BL. To confirm the wavelength-specificity, the cells were further exposed to red (RL), purple (PL), and white light (WL). BL and WL exposure increased ROS levels, but only BL exposure led to apoptosis in primary MCs. Thus, BL accounted for the harmful part of WL exposure. When cells were simultaneously exposed to BL and RL (i.e., PL), cell damage due to BL could be partly prevented, as could the inhibition of p53, demonstrating the protective effect of RL and p53 dependency. In contrast, BL hardly induced apoptosis in MIO-M1 cells, which is likely due to the immortalization of the cells. Therefore, enhanced oxidative stress levels can significantly harm MC function, probably leading to decreased retinal survival and, thus, further enhancing the progression of retinal diseases. Preventing the cell death of these essential retinal cells represents a promising therapy option to enhance retinal survival.

## 1. Introduction

The length of time spent indoors and in front of screens has increased drastically over the last few years, mainly due to enhanced remote working since the SARS-CoV-2 pandemic, resulting in longer and more intense exposure to artificial light [1]. Especially with the development and usage of light-emitting diodes (LEDs), which have a relatively high blue light (BL) emission, BL exposure has become incredibly common in modern environments. Nevertheless, it is important, to note that sunlight is still the greatest source of BL exposure. However, BL from yellow phosphor LEDs has a peak emission at ~450 nm ± 35 nm, which is distinct from that of the daylight spectrum [2], thus we cannot exclude the possibility that artificial BL has specific impacts on the eye. Additionally, BL emission from white LEDs appears to increase over the lifetime of the LED [3]. BL exposure is well known to contribute to computer vision syndrome caused by the almost constant use of LEDs in monitors, smartphones, computers, and other appliances [4]. Due to its short wavelengths (400–500 nm), BL has a high photochemical energy. Despite containing less energy than UV light, it can cause significant damage to the retina as it can pass through the eye almost unfiltered [5,6]. This is especially dangerous when the eye is still young and, therefore, unclouded, as the amount of light reaching the retina is decreased with increasing age due to pupil size reduction (senile miosis) and yellowing of the crystalline lens [7]. In contrast, over 65% of BL at 460 nm is transmitted to the retina in children younger than 9 years old [8]. The impact of screen reading on schoolchildren’s visual acuity further revealed that screen reading can lead to the development of poor eyesight and a higher incidence of nearsightedness [9]. This was confirmed in recent studies in rhesus monkeys, where the authors showed that short-wave light, such as BL, is more likely to lead to myopia [10]. Thus, the overuse of BL-emitting LEDs at a young age can harm the retina and lead to early retinal diseases. Jaadane et al. demonstrated that most of the currently used LEDs are toxic to the retina, likely due to the effects of shorter wavelengths on the mitochondria [11]. In particular, the exposure of retinal pigment epithelium cells (RPEs) to currently used gallium nitride (GaN)-based LEDs affects the mitochondria more than classical LEDs [11]. They, therefore, criticized that the photosensitivity threshold in rats is largely overestimated, possibly making the use of some current LEDs and monitors dangerous. In retinal cells, low BL intensities of smartphone displays are already sufficient to enhance oxidative stress levels and apoptosis [12]. 

As multiday exposure to BL could increase load on cellular repair mechanisms, it is, furthermore, likely that the accumulation of subthreshold damage to cellular processes produces long-term sub-apoptotic cell stress that finally leads to apoptosis, associated with progressive diseases such as age-related macular degeneration (AMD) [13]. In this context, BL can harm the retina via ROS accumulation and DNA damage, especially when the repair mechanisms of the eye for BL weaken due to aging [14,15]. The extent of damage can be even greater if crystalline lenses are removed by cataract surgery, allowing more BL to reach the retina [16]. The combination of light exposure, elevated metabolic activity, accumulation of oxidized lipoproteins, and decreased antioxidant functions during aging make retinal tissues even more vulnerable to oxidative stress [17,18]. Therefore, a potential role of BL in the development of progressive oxidative stress-based retinal diseases needs to be investigated.

BL damage is characterized by the excessive production and accumulation of free oxygen radicals (reactive oxygen species, ROS) in the mitochondria [19], further overcoming the existing protective mechanisms [15,20]. ROS include superoxide anion (O_2_^−^), hydroxyl radical (^•^OH), hydrogen peroxide (H_2_O_2_), nitric oxide (NO), and singlet oxygen (O_2_). Hydroxyl radicals, the most reactive oxygen form, can form out of H_2_O_2_ [21]. ROS production mainly results from the mitochondrial respiratory chain, but also due to photochemical and enzymatic reactions and as a result of light exposure or ionizing radiation. In general, ROS can cause DNA damage, such as double-strand breaks [22], and further induce epigenetic changes resulting in atypical gene expression, if the damage is not lethal [23]. This, and numerous other damaging effects, can lead to pathological events such as inflammation, autophagy [24], and endoplasmic reticulum stress [25], likely resulting in apoptosis. 

In ophthalmology, oxidative stress is suspected to be involved in the development of multiple eye diseases such as senile cataract [26], AMD [27], uveitis [28], premature retinopathy [29], and ocular inflammation [30]. In the case of AMD, a positive feedback loop of oxidative stress and inflammation seems to be involved in the pathogenesis [23]. Not all retinal cells are similarly susceptible to light damage; inner retinal glia cells, such as Müller cells (MC), are almost transparent in contrast to RPEs. They, therefore, lack a photosensitizer such as melanin or lipofuscin, which make RPEs vulnerable to photochemical damage [31]. Thus, MCs are not yet known to be directly involved in phototoxicity [32]; however, it was previously shown that the MCs of BL-exposed rat retinas demonstrated hypertrophy and increased GFAP expression [32]. Enhanced GFAP expression, a marker of gliosis, is considered to be a hallmark of retinal stress, resulting in degenerative alteration of the inner retina and neurodegeneration in chronic diseases such as AMD, glaucoma, or diabetic retinopathy [32,33]. As a response to acute and transient stimuli, such as enhanced oxidative stress, MCs secrete antioxidant and protective factors, but when stress becomes chronic, they start secreting tumor necrosis factor (TNF) or different interleukins, thus inducing inflammation and making them additional stressors for neuronal cells [34]. AMD-affected retinas are also characterized by an increased immune reactivity [35], a circumstance to which activated, pro-inflammatory MCs could also contribute. It is known, that MCs, which are usually very linear, start to lose their shape in AMD and begin to extend and migrate through the retina, resulting in the formation of a membrane below or on top of the retina [36]. These membranes could block drug therapies from reaching their planned targets. Furthermore, when the remodeled MCs exit the retina, they interact with cells they are normally separated from, such as the RPEs, where the AMD disease is progressing. Therefore, damaged MCs could also negatively influence RPEs, probably resulting in a dysfunction of these cells and thus further promoting AMD progress. 

In general, MCs are the only cells spanning the entire retinal thickness, generating numerous contacts to neighboring retinal cells. For guinea pigs, it is known that they act as wavelength-dependent optical fibers and leak blue–violet light toward the surrounding tissue [37]. Therefore, these cells naturally come in contact with BL when it comes to light guidance. The remodeling of MCs early in diseases could push inflammation and oxidative stress levels, as the majority of retinal diseases are associated with gliosis of MCs [38], and nearly all retinal cells are dependent on MCs. Taken together, this indicates an important role for MC in BL-induced oxidative stress-based retinal degeneration.

## 2. Results

### 2.1. Single Blue Light Exposure Significantly Enhanced Oxidative Stress Levels in Müller Cell-Derived Cells and MIO-M1 Cells 

Since oxidative stress is a hallmark of many neurodegenerative diseases [21,26,39], the induction of oxidative stress in primary Müller cell-derived cells (MCs) and human MIO-M1 cells after BL exposure was evaluated via hydrogen peroxide (H_2_O_2_) measurements (Figure S1). Cells were exposed to BL with an intensity of 30 mW/cm^2^ for 1 or 2 h and further cultivated for 6 or 24 h. A significantly induced H_2_O_2_ level was detected in primary MCs and human cell lines, with the strongest increase at 2 h of exposure followed by 6 h of cultivation (Figure S1; MCs: +955%; MIO-M1: +11,430%), proving that BL exposure caused enhanced H_2_O_2_ levels. Remarkably, H_2_O_2_ levels were very low in the control groups of MIO-M1 cells compared to primary MCs (at 6 h: −89% in MIO M1 cells; at 24 h: −79% in MIO-M1 cells) (Figure S1). The significant induction of H_2_O_2_ levels even after 24 h in MCs (1 h BL: +1.2-fold ± 0.25; 2 h BL: +0.796-fold ± 0.25) (Figure S1A) and MIO-M1 cells (1 h BL: +6.76-fold ± 1.24; 2 h BL: +36.68-fold ± 3.61) (Figure S1B) strongly suggests a high susceptibility of these cells toward BL.

### 2.2. Blue Light Induced Apoptosis in MCs, but Not in MIO-M1 Cells

**Figure 1:**
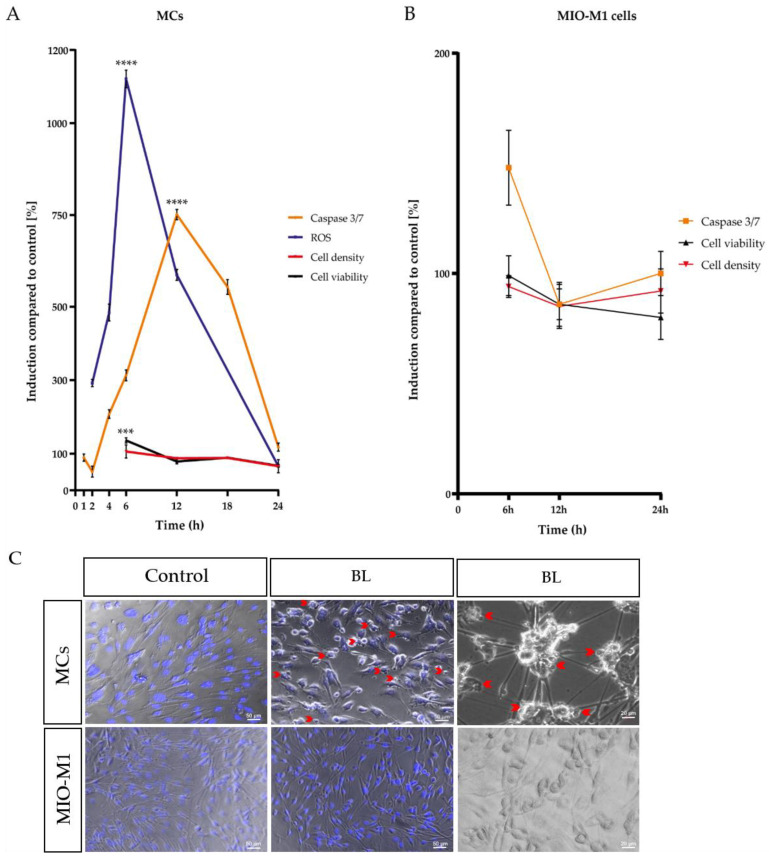
Time course of blue light (BL)-induced processes in Müller cell-derived cells and human MIO-M1 cells. Caspase 3/7 activity, cell viability, and cell density were measured after 1.5 h BL exposure in (**A**) Müller cell-derived cells (MCs) over time compared to a 100% set control. H_2_O_2_ levels increased rapidly with a peak after 6 h, followed by enhanced caspase 3/7 activity with a peak at 12 h. Cell viability and density decreased after 24 h, indicating apoptosis as the primary cell death pathway. (**B**) MIO-M1 cells only had a slight increase in caspase 3/7 activity after 6 h. Cell viability and density levels were similar to control levels 24 h after exposure. (**C**) Morphological analysis revealed apoptotic blebbing (red arrows), cell shrinkage, and condensed nuclei (Hoechst staining: blue) in BL-exposed MCs 24 h after exposure (compared to unexposed control). In contrast, BL exposure did not induce apoptotic blebbing or condensed nuclei in MIO-M1 cells. Bar graphs represent the mean values and SEM. The control was set to 100%, and induction was compared to control levels. Original data are provided in Table S1; *n* = 5–15, repeated three times. Statistical differences are indicated as follows: *** *p* < 0.001, and **** *p* < 0.0001 compared to control, according to Welch’s one-way ANOVA.

As MCs exhibited a pronounced sensitivity to BL, it is crucial to investigate the consequences of the high H_2_O_2_ levels on these cells. To this end, caspase-mediated apoptosis, metabolic activity, cell viability, and cell density were measured in both primary MCs and the human MIO-M1 cell line over 24 h (Figure 1A,B).

The analysis of a single-time exposure of 1.5 h revealed significantly increased H_2_O_2_ levels in MCs with a peak at 6 h after exposure (+11.21-fold ± 0.61, *p* < 0.0001) (Figure 1A). In addition, a significantly enhanced caspase 3/7 activity with a peak at 12 h (+7.182-fold ± 0.3453, *p* < 0.0001) after exposure was detected (Figure 1A). In accordance with the prior two observed effects, the metabolic activity, determined via MTS assay, was slightly increased after 6 h (+21%, *p* < 0.001) and did not decrease before 24 h (−40%, *p* < 0.0001), further pointing to the highly energy-consuming death process, apoptosis, as the primary cell death pathway (Figure 1A). This hypothesis was again corroborated by the decreased cell density of BL-exposed MCs 24 h after exposure (−0.58-fold ± 0.04), determined by crystal violet staining (Figure 1A). During morphological analysis, condensed nuclei, apoptotic blebs, and cell shrinkage were observed in MCs (Figure 1C), again indicating apoptosis as the primary cell death pathway for MCs. In contrast to BL exposure of the primary MCs, BL exposure of MIO-M1 cells caused less caspase 3/7 induction after 6 h of cultivation (281% less than in MCs) (Figure 1B). The observed induction in caspase 3/7 activity (+50%) decreased further toward control levels after 24 h (Figure 1B). Likewise, there was no strong decrease in cell viability or density 24 h after BL exposure (Figure 1B). Morphologically, MIO-M1 cells did not show apoptotic blebs (Figure 1C) and also demonstrated almost no cell shrinkage or cell death 24 h after BL exposure (Figure 1C). Taken together, the human MIO-M1 cell line was less sensitive to BL compared to primary MCs.

As many lines of evidence point toward apoptosis as the main mechanism behind BL-induced damages to MCs, an apoptosis signaling array was performed with cell lysates of unexposed and exposed (1.5 h BL, 30 mW/cm^2^) cells with 6 and 12 h further cultivation (Figure S2). This semi-quantitative detection revealed an increase in pro-apoptotic proteins such as p53 (1.42-fold increase after 12 h, compared to control), TNFα (4.82-fold increase after 12 h, compared to control), BID and BIM (1.65- and 1.62-fold increase after 12 h, compared to control), and caspase 3 (1.77-fold increase after 12 h, compared to control) (Figure S2). Furthermore, 12 h after exposure, BAX expression was 1.33-fold increased, but BCL-2 expression was decreased (−27%), implying an increase in free pro-apoptotic BAX after BL exposure (Figure S2). Therefore, an overall increase in pro-apoptotic protein expression can be attributed to BL exposure in MCs.

### 2.3. Particular Blue Light in White Light Artificial Exposure Harms Müller Cell-Derived Cells

**Figure 2:**
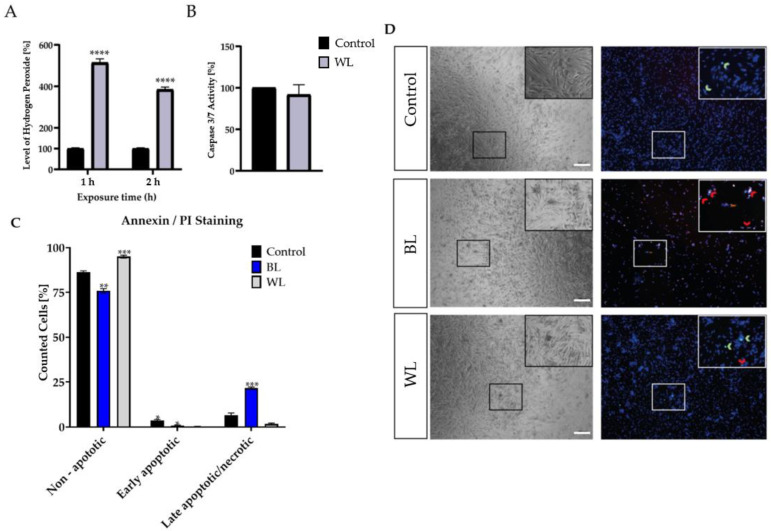
Blue light exposure enhanced cell death in Müller cell-derived cells in contrast to white light. (**A**) H_2_O_2_ levels in white light (WL, 30 mW/cm^2^)-exposed porcine Müller cell-derived cells (MCs) were significantly increased after 1 or 2 h of WL exposure. (**B**) MCs showed no increase in caspase 3/7 levels 6 h after WL exposure; *n* = 10, three independent experiments; controls were set to 100%. (**C**) BL- or WL-exposed MCs were stained with annexin V/PI and demonstrated significantly more (late) apoptotic cells compared to unexposed cells. (**D**) Representative pictures of annexin/PI staining displayed increased PI (red) and annexin V (green) staining in BL-exposed MCs 24 h after exposure. Less PI and annexin V staining was observed in WL-exposed MCs. Red arrows indicate late apoptosis/necrosis, while green arrows indicate early apoptosis; *n* = 15, analyzed using ImageJ. Bar graphs represent mean values as percentages and the SEM. Statistical differences are indicated as follows: * *p* < 0.05, ** *p* < 0.01, *** *p* < 0.001, and **** *p* < 0.0001 compared to the control, according to Welch’s Student‘s *t*-test or Welch’s one-way ANOVA. Scale bar = 200 μm.

Over the last few decades, more and more time has been spent indoors and in front of screens, thus increasing artificial WL exposure. LEDs contain a high amount of harmful BL, but also protective wavelengths such as RL [40,41,42]. To investigate whether the proportion of BL in a single WL exposure is enough to harm MCs, and whether the proportion of RL is enough to protect the MCs, MCs were exposed to WL with an intensity of 30 mW/cm^2^ (Figure 2). In parallel, MCs were exposed to BL with the same intensity to reveal its toxic effect (Figure 2). BL-enriched WL was sufficient to induce significantly oxidative stress levels in MCs (Figure 2A). However, a single WL exposure did not induce caspase 3/7 activity (Figure 2B). To verify that a single WL exposure did not induce apoptosis, PI (propidium iodide, red) and annexin V/FITC (green) staining was conducted (Figure 2C,D). Annexin V/DAPI staining that does not colocalize with PI represents early apoptotic cells, whereas annexin V/PI colocalization represents late apoptotic or necrotic cells. Exposure to BL resulted in increased cell death, as indicated by higher annexin V/PI staining, in contrast to WL exposure (Figure 2C,D), where no difference to the controls was observable. This finding indicates that a single WL exposure did not lead to cell death in MCs, whereas BL exposure did. Thus, BL constitutes the harmful part of WL exposure.

**Figure 3:**
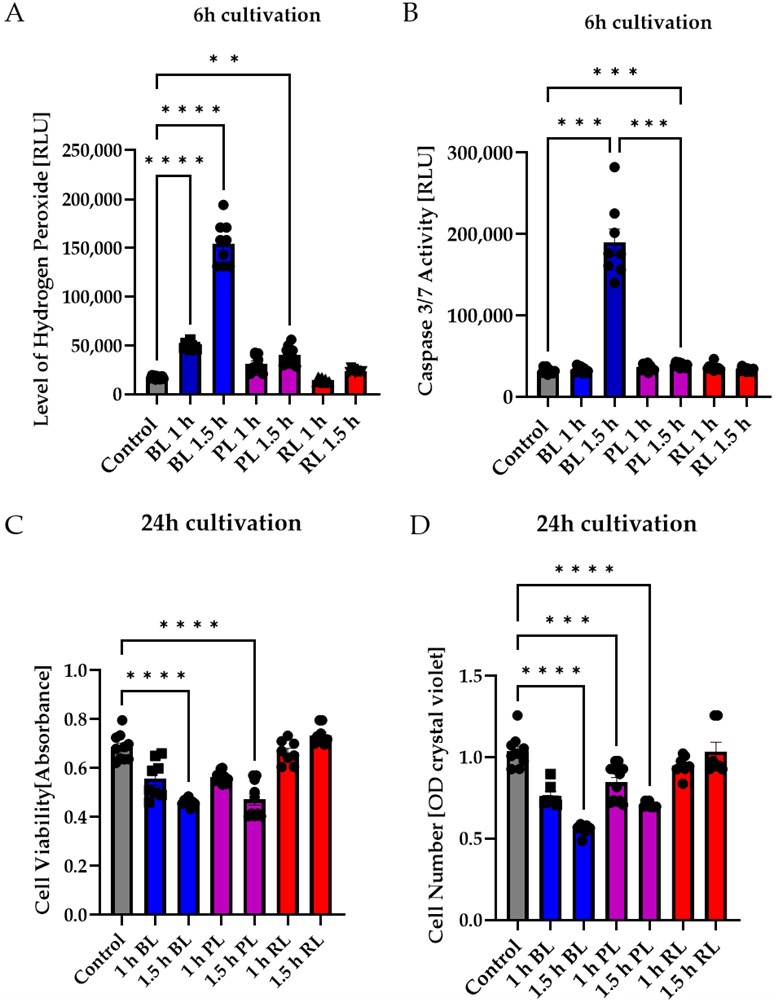
Blue light exposure, in particular, led to apoptosis in primary Müller cell-derived cells, whereas red light had no toxic effect. Müller cell-derived cultures (MCs) were exposed to blue light (BL, 15 mW/cm^2^), purple light (PL, a combination of blue and red light, 15 mW/cm^2^), or red light (RL, 630 nm, 3 mW/cm^2^) for 1 or 1.5 h and further cultivated for 6 or 24 h. (**A**) Oxidative stress levels were significantly induced 6 h after BL or PL exposure, but not RL exposure. (**B**) Caspase 3/7 activity was slightly induced due to PL exposure and strongly induced after BL exposure. (**C**) Cell viability significantly decreased 24 h after BL and PL exposure. Again, RL exposure did not affect MCs. (**D**) BL and PL exposure led to a significant and strong decrease in cell numbers 24 h after exposure, whereas RL exposure did not affect MC cell numbers. Bar graphs represent the mean values and SEM. The experiments were repeated three times with similar results. Statistical differences are indicated as follows: ** *p* < 0.01, *** *p* < 0.001, and **** *p* < 0.0001 compared to control, according to Welch’s one-way ANOVA.

To further evaluate whether the amount of BL in WL exposure in particular leads to apoptosis induction of MCs, MCs were exposed to BL or PL (15 mW/cm^2^), which resembled a combination of BL and red light (RL, 630 nm) in our exposure set up, for 1 or 1.5 h (Figure 3). Furthermore, pure RL (630 nm, 3 mW/cm^2^) exposure was performed to evaluate the non-toxic effect of this wavelength on MCs (Figure 3). RL is known to reduce oxidative stress levels and could, therefore, protect against the induction caused by BL exposure [41]. In a first step, oxidative stress levels were measured 6 h after exposure (Figure 3A). The highest oxidative stress level was observed after 1.5 h of BL exposure, while PL exposure (1 and 1.5 h) also significantly induced oxidative stress (1 h: +1.99-fold, *p* < 0.05; 1.5 h: +2.577-fold, *p* < 0.001; Figure 3A). In contrast, RL exposure did not increase oxidative stress levels (Figure 3A). It can be concluded that, in WL exposure, BL induces oxidative stress levels, but not RL. Furthermore, the RL in the PL exposure resulted in less oxidative stress; hence, RL potentially counteracts harmful BL exposure. To evaluate if the BL amount in PL exposure was enough to induce apoptosis, caspase 3/7 activity was determined 6 h after exposure (Figure 3B). Compared to BL exposure (+6.0-fold, *p* < 0.001), only 1.5 h of PL exposure was able to slightly induce caspase 3/7 activity in MCs (Figure 3B; +1.22-fold, *p* < 0.001). All other conditions had no effect on caspase 3/7 activation. This finding further underlines the assumption that only BL can significantly and strongly induce apoptosis in MCs, while parallel exposure to RL significantly (*p* < 0.001) decreased caspase 3/7 activity (Figure 3B). In this context, 1.5 h of BL or PL exposure led to a significant (*p* < 0.0001) decrease in cell viability 24 h after exposure (BL: −28%, PL: −22%), but RL exposure did not (Figure 3C). Furthermore, BL exposure, in particular, significantly decreased cell density 24 h after exposure (1.5 h BL: −55%, *p* < 0.0001) (Figure 3D). PL exposure also reduced cell density (1 h PL: −0.15%, *p* < 0.001; 1.5 h PL: −35%, *p* < 0.0001), but to a lesser degree than BL exposure (Figure 3D). Furthermore, neither 1 or 1.5 h of RL exposure altered cell density (Figure 3D). To further clarify that BL induced apoptosis in MCs, the ATP content in MCs 6 h after 1.5 h exposure of BL, PL, or RL was investigated (Figure S3). BL and PL exposure slightly decreased ATP content (BL: −10%, PL: −8%; Figure S3). Due to only a slight decrease being observed instead of a strong and rapid ATP loss as seen in necrosis, this finding further underlines apoptosis as the BL-induced cell death pathway. Importantly, RL exposure did not alter ATP content (Figure S3).

### 2.4. MIO-M1 Cells Demonstrate Robustness to Blue, Purple, and Red Light Exposure

**Figure 4:**
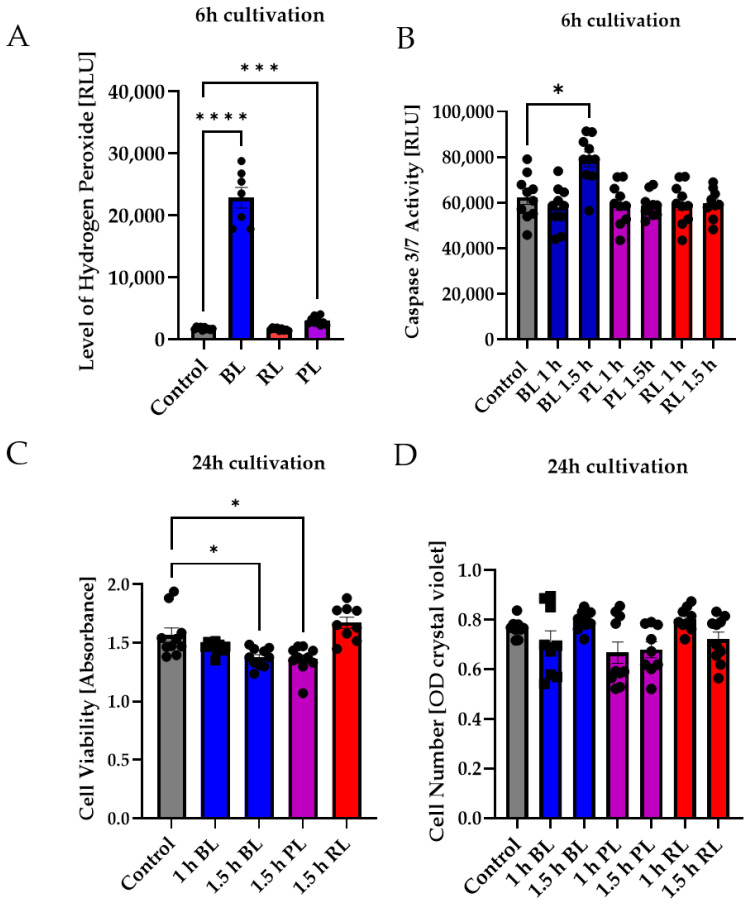
Neither purple nor red light exposure strongly affected MIO-M1 cells. Human MIO-M1 cells were exposed to blue (BL), purple (PL), or red light (RL) for 1.5 h and further cultured for 6 or 24 h. (**A**) Oxidative stress levels were significantly induced 6 h after BL or PL exposure, but not after RL exposure. (**B**) Only BL exposure led to significantly increased caspase 3/7 activity in MIO-M1 cells 6 h after exposure. (**C**) Cell viability was slightly but significantly decreased 24 h after 1.5 h of BL or PL exposure. (**D**) BL, PL, and RL exposure did not alter cell numbers 24 h after exposure. Bar graphs represent the mean values and SEM. The experiments were repeated three times with similar results. Statistical differences are indicated as follows: * *p* < 0.05, *** *p* < 0.001, and **** *p* < 0.0001 compared to control, according to Welch’s one-way ANOVA or Kruskal–Wallis test.

In the next step, the effect of PL and RL exposure on human MIO-M1 cells was investigated to determine differences between primary cells and the human cell line. Again, BL induced oxidative stress in MIO-M1 cells (1.5 h exposure, 6 h cultivation), whereas PL exposure only slightly induced oxidative stress levels, and RL exposure did not alter stress levels at all (Figure 4A). PL exposure (1 and 1.5 h) did not affect caspase 3/7 activity in MIO-M1 cells (Figure 4B), while BL only slightly induced caspase 3/7 activity (+27%, *p* < 0.05, Figure 4B). RL did not alter caspase 3/7 activity (Figure 4B). The cell viability of MIO-M1 cells only slightly decreased following 1.5 h BL or PL exposure (−16% and −15%, respectively, *p* < 0.01; Figure 4C). In contrast, RL exposure slightly (but not significantly) increased viability. BL, PL, and RL exposure had no effect on MIO-M1 cell numbers 24 h after exposure (Figure 4D).

Taken together, BL increased oxidative stress levels in MIO-M1 cells, but PL exposure only slightly induced oxidative stress levels and was, therefore, not able to alter caspase 3/7 activity, cell viability, or cell density (Figure 4). RL exposure did not affect MIO-M1 cells at all (Figure 4). In summary, MIO-M1 cells did not react as primary MCs to our applied exposure. Because MIO-M1 cells generally demonstrated a very slight effect, only investigations on porcine MCs were performed further.

### 2.5. Blue Light-Induced Apoptosis in Müller Cell-Derived Cells Can Be Prevented by Inhibition of p53

**Figure 5:**
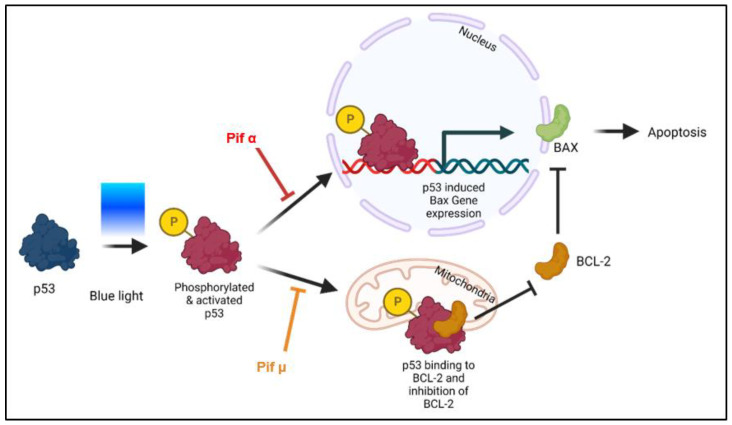
Blue light-induced apoptosis can be prevented through p53 inhibition (model). Although direct effects have not yet been shown, it is likely that following blue light (BL) exposure, the tumor suppressor protein p53 is modified (e.g., phosphorylated) and, thus, accumulates in an activated form in the cell, resulting in expression of its pro-apoptotic target genes such as *Bax*. Based on current knowledge, pifithrin α (Pif α), a cell-permeable transcriptional p53 inhibitor, inhibits p53-dependent gene transcription. Therefore, Pif α could prevent *Bax* expression after BL exposure. It is further known that pifithrin µ (Pif µ) prevents p53 function on the mitochondrial side without affecting p53 transcriptional activities. Activated and accumulated p53 normally binds to BAX inhibitor BCL-2, thus resulting in an increase in free BAX and induced apoptosis. Therefore, Pif µ treatment could block the binding of p53 to BCL-2, resulting in an increase in free BCL-2 and, therefore, less free pro-apoptotic BAX. Pif µ could, therefore, also inhibit BL-induced apoptosis.

**Figure 6:**
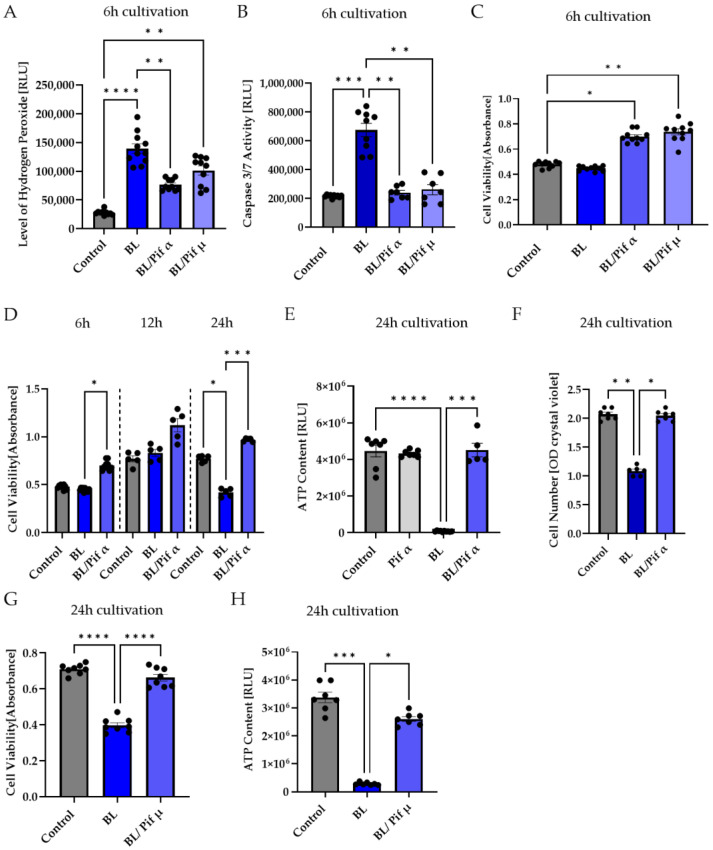
Inhibition of p53 reduced apoptosis in porcine Müller cell-derived cells. (**A–H**) MCs were exposed to blue light (BL, 30 mW/cm^2^) for 1.5 h and further cultivated for the indicated timepoints. Treatment with 1 μM pifithrin α (Pif α) or 10 μM pifithrin µ (Pif µ) immediately after exposure resulted in (**A**) decreased BL-induced oxidative stress levels 6 h after exposure, but only treatment with Pif α was able to significantly reduce the level of hydrogen peroxide. (**B**) A significantly reduced caspase 3/7 activity back to control levels was determined with each inhibitor. (**C**) Cell viability in MCs was enhanced by Pif α or Pif µ treatment. (**D**) Pif α incubation after BL exposure resulted in significantly induced cell viability compared to BL-exposed cells in a time-dependent manner. Cell viability was always higher compared to unexposed controls. Each timepoint represents an independent experiment. (**E**) The strong decrease in ATP content in MCs 24 h after BL exposure could be rescued by immediate treatment with Pif α. Treatment of unexposed MCs with Pif α did not alter ATP content. (**F**) Pif α treatment could rescue significantly decreased cell numbers 24 h after BL exposure. (**G**) Cell viability was also significantly enhanced due to treatment with Pif µ, compared to BL-exposed MCs. (**H**) Evaluation of ATP content revealed a significant increase due to Pif µ treatment 24 h after BL exposure, compared to BL-exposed MCs. Bar graphs represent the mean values and SEM. The experiments were repeated three times with similar results. Statistical differences are indicated as follows: * *p* < 0.05, ** *p* < 0.01, *** *p* < 0.001, and **** *p* < 0.0001 compared to control/BL, according to Welch’s one-way ANOVA or Kruskal–Wallis test.

Since the tumor suppressor protein p53 is the main regulator concerning cell death and induces apoptosis in highly stressed cells, we hypothesized that the inhibition of p53 would prevent BL-induced apoptosis in MCs. In the first step, the effect of pifithrin α (cyclic pifithrin-α-*p*-nitro), a small, selective posttranscriptional inhibitor of p53, and pifithrin µ, a mitochondrial inhibitor of p53, was evaluated (Figure 5 and Figure 6).

Only Pifithrin α treatment right after BL exposure (1.5 h) significantly decreased BL-induced oxidative stress levels after 6 h (−53% compared to BL exposure, *p* < 0.01) (Figure 6A), demonstrating that BL-induced p53 levels probably resulted in the expression of pro-oxidants, which could be prevented by pifithrin α treatment, but not by pifithrin µ. In contrast to pifithrin α, pifithrin µ is only able to block the p53 mitochondrial binding to antiapoptotic BCL-2 (Figure 5); therefore, p53-dependent gene expression still takes place. Nevertheless, treatment with either p53 inhibitor resulted in significantly less caspase 3/7 activity 6 h after BL exposure (pifithrin α: −65%, *p* < 0.0001; pifithrin µ: −62%, *p* < 0.0001) (Figure 6B), proving that both inhibitors are able to protect the cells from BL-induced apoptosis. Furthermore, this suggests that BL-induced p53 activity mainly results in expression of pro-apoptotic BAX, because pifithrin µ is only able to reduce the level of free BAX and has no known effect on other pro-apoptotic proteins. Furthermore, cell viability was increased 6 h after BL exposure by both inhibitors (Figure 6C).

Treatment with pifithrin α increased the cell viability of BL-exposed MCs back to control levels, further increasing 12 and 24 h after exposure (Figure 6D), and rescued the decrease in MC cell numbers 24 h after BL exposure back to control level (Figure 6F). Interestingly, 24 h after BL exposure, the ATP content of exposed MCs dropped to almost zero, probably due to secondary necrosis (Figure 6E). Treatment with pifithrin α rescued ATP levels back to control levels (Figure 6E).

To determine if pifithrin µ treatment is as effective as pifithrin α concerning cell viability and ATP levels, these parameters were also examined 24 h after BL exposure (Figure 6G,H). Pifithrin µ treatment was found to be as protective as pifithrin α, further supporting the theory that cell death proceeds mitochondrially. Due to the longer half-life of cyclic pifithrin α (compared to non-cyclic pifithrin α), the broader available information, and the significant reduction in oxidative stress levels after BL exposure, subsequent studies concentrated on pifithrin α.

**Figure 7:**
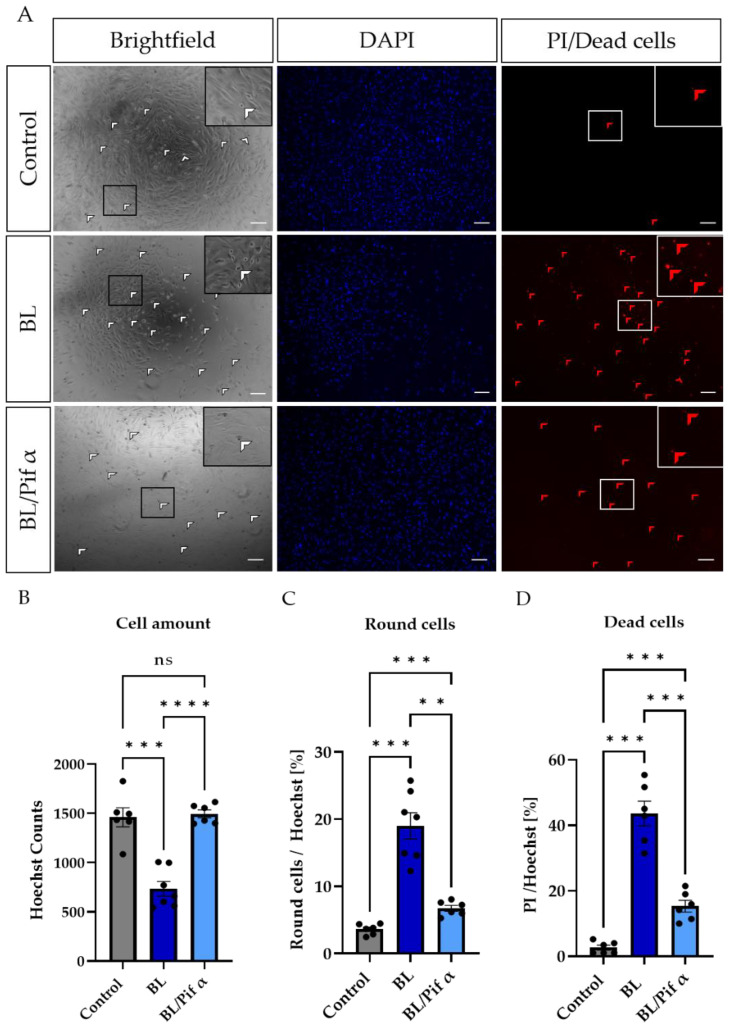
Treatment with pifithrin α significantly reduced apoptotic cell death in primary Müller cell-derived cells. Müller cell-derived cells (MCs) were exposed to BL (30 mW/cm^2^) for 1.5 h and further cultivated for 24 h. MCs were treated with 1 µM pifithrin α immediately after exposure. (**A**) Bright-field images revealed more round cells (white arrows) in BL-exposed MCs, with a reduction in number following pifithrin α treatment. Moreover, BL induced more PI-positive (thus dead) cells (red arrows), with this number again reduced by pifithrin α. (**B**) MCs were stained with Hoechst to quantify the cell amount per condition. Significantly fewer cells were counted after BL exposure; however, due to pifithrin α treatment, an equal number of cells to the control were observed. (**C**) Treatment with pifithrin α strongly reduced the number of round cells after BL exposure. The counted number of round cells was normalized by Hoechst staining in each sample (round cells/Hoechst) to show the percentage of round cells. (**D**) Significantly more PI-positive cells (i.e., dead cells with ruptured membranes) were observed following BL exposure. Treatment with pifithrin α decreased the number of PI-positive cells. The number of PI-positive cells was normalized by Hoechst staining in each sample (PI/Hoechst) to demonstrate the percentage of dead cells. *n* = 6/condition, analyzed using ImageJ. Bar graphs represent the mean values and SEM. Statistical differences are indicated as follows: ns: not significant, ** *p* < 0.01, *** *p* < 0.001, and **** *p* < 0.0001 compared to control/BL, according to Welch’s one-way ANOVA. Scale bar: 200 µM.

**Figure 8:**
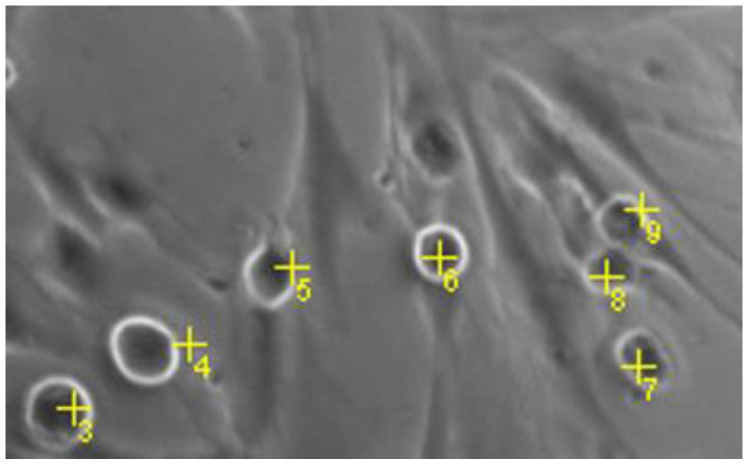
Quantification of round cells. Numbers representing cell count of round cells.

Morphologically, more rounded cells (Figure 8) were observed in BL-exposed MCs (Figure 7A,C), further underlining apoptosis as the primary cell death pathway. Treatment with pifithrin α significantly reduced the number of rounded cells (Figure 7A,C) and increased the general number of MCs (as indicated by Hoechst staining; Figure 7A,B). In this context, the number of dead cells (PI-positive cells) was also significantly reduced following treatment with pifithrin α right after BL exposure (Figure 7A,D).

In summary, these data strongly suggest a role for p53 in BL-induced apoptotic processes in primary MCs.

## 3. Discussion

In many retinal diseases, such as AMD, the involvement of MCs is poorly understood as they are not the primary trigger of diseases. Nevertheless, it is undisputed that they play an essential role in the retina and its survival, and that their dysfunction or death can severely affect the retina. Different retinal diseases are based on oxidative stress, such as AMD, glaucoma, or diabetic retinopathy (DR) [43]. There are various approaches to experimentally induce oxidative stress and, thus, retinal damage, e.g., by administering H_2_O_2_ [44] or cobalt chloride [45,46]. A more natural variant is BL exposure, as artificial light (as well as sunlight) contains high amounts of BL, and it is able to induce oxidative stress and retinal cell death. However, previous work has hardly addressed whether BL also has a damaging effect on MCs and how this damage occurs molecularly. MCs, in contrast to RPEs, lack retinal chromophores such as melanin or lipofuscin, which generate various types of ROS when irradiated with visual light [47,48]. Thus, it was a general thought that these cells would react less sensitively to BL exposure. However, human MIO-M1 cells express various opsins, i.e., short-wave (blue) sensitive cone (S-) opsins and melanopsin. Stimulation with 480 nm predominantly activates melanopsin, and the cytosolic calcium responses detected due to short-wave exposure might be induced by the activation of opsins in these cells [49]. Furthermore, primary chicken MCs express non-visual opsins (i.e., neuropsin), and BL exposure (480 nm) significantly increased the expression levels and nuclear localization. Likewise, intracellular calcium increase was detected after BL exposure [50]. It is further known that BL exposure induces the aggregation of S-opsin, resulting in severe cell damage [51]. Therefore, BL exposure could lead to cell death via opsins in MCs. Mitochondria are also able to absorb visual light, as their flavins and cytochrome c oxidases are able to absorb different wavelengths in distinct ways. In ARPE-19 cells, also lacking retinal chromophores, BL damage seems to be related to effects on mitochondrial pigments [52]. Mitochondrial cytochrome c oxidase absorbs BL maximally around 400–410 nm [52], whereas (ribo)flavin demonstrates an absorption maximum at 420–520 nm and induces protein oxidation, as well as H_2_O_2_ generation [53,54]. Since we applied BL irradiation in the wavelength range 455–465 nm and also observed an enormous increase in H_2_O_2_ (Figure S1), it can be assumed that the damage induced by BL in MCs was induced mainly by mitochondrial flavins. The induction of caspase 3/7 indicated apoptosis as the primary cell death pathway, which was also supported by the fact that cell viability only decreased after 24 h (Figure 1). Since apoptosis is an active process with cellular metabolism maintained throughout the entire phase, an increase in or stability of metabolic activity at an early timepoint followed by a low signal 24 h after exposure is consistent with the induction of apoptosis, whereas a rapid decrease is more indicative of necrosis [55]. This assumption was further confirmed with ATP measurements 6 h after BL exposure demonstrating only a slight loss of ATP (Figure S3). This is in accordance with the study of H_2_O_2_-incubated primary rat MCs, displaying an increased number of floating and apoptotic MCs [56]. BL exposure of MCs also led to enhanced expression of numerous pro-apoptotic proteins in a screen for apoptotic proteins (Figure S2), underlining apoptosis as the primary cell death pathway. Likewise, BL-exposed MCs demonstrated significant cell shrinkage and blebbing (Figure 7A,C). About half of the exposed cells were PI-positive, thus demonstrating a ruptured cell membrane as known from secondary necrosis (Figure 7A,D). This goes hand in hand with the finding that, without sufficient clearance, secondary necrosis occurs after apoptosis, resulting in a loss of cell membrane integrity. ATP measurements 24 h after BL exposure revealed almost a total loss of ATP in MCs, thus confirming secondary necrosis (Figure 6E). The fact that the drop in ATP was this extreme 24 h after BL exposure could also be partly due to the cell loss occurring as a result of completed apoptosis (~60% of the cells) (Figure 1A). Annexin V/PI staining on BL-exposed MCs demonstrated an increased number of late apoptotic/necrotic cells after 24 h, but fewer early apoptotic cells compared to untreated cells (Figure 2C,D). This further indicates secondary necrosis due to the insufficient clearance of apoptotic cells. In vivo, secondary necrosis causes inflammation; thus, secondary necrosis in MCs after oxidative stress-induced apoptosis could enhance retinal inflammation and speed up the progress of retinal diseases. In this context, it is important to mention that epidemiological studies evaluating (BL) exposure and the risk of developing AMD have several limitations. Firstly, AMD resembles a very complex disease and exposure to light over a lifetime cannot be determined accurately. Secondly, genetic variability, lifestyle, and patient recall concerning the exposure to (blue) light could disturb the true relationship between exposure and AMD development. It is even more difficult to evaluate the real-life impact of BL-damaged MCs in AMD development, as the retina is a complex tissue, and MCs only make up a very small amount of the cell population of the retina (~1.5% of murine retina [57]). Nevertheless, on the basis of our data, MCs demonstrate a high sensitivity to BL exposure and enhanced oxidative stress levels, probably due to a lack of oxidative stress defense, making them early targets in the development of oxidative stress-based retinal diseases. To further evaluate whether apoptosis induction in MCs by enhanced oxidative stress levels was dependent on BL (and, thus, wavelength), MCs were further exposed to PL (which combines RL and BL exposure), RL, and WL (Figure 2 and Figure 3). RL exposure did not increase oxidative stress levels in MCs, but PL and WL exposure did, leading to the conclusion that BL is the harmful, oxidative stress-inducing part of artificial (WL) exposure (Figure 2 and Figure 3). Furthermore, PL exposure resulted in enhanced caspase 3/7 activity and less cell viability/density in MCs (Figure 3). However, the induction of apoptosis was lower compared to BL exposure (Figure 3). In contrast, RL exposure did not alter MCs, and, because PL combines harmful BL with non-toxic (and probably protective long wavelength) RL, RL exposure probably counteracts BL exposure and reduces BL-induced retinal damage. This would also explain why single WL exposure did not result in MC death (Figure 2B–D). Likewise, under in vivo situations, short exposure to sunlight (or artificial light) does not result in retinal damage. Thus, accumulated or prolonged exposure to BL-containing WL might lead to the development or progression of oxidative stress-based retinal diseases. In ARPE-19 cells, RL, delivered after BL exposure, is significantly blunted [42]. Accordingly, RL could have therapeutic use to counteract the retinal cell damage that likely happens during retinal diseases such as AMD. It is also conceivable that repeated RL irradiation increases viability and resistance to oxidative stress in MCs. Further studies are, therefore, crucial to evaluate the protective effect of RL irradiation after BL exposure.

Furthermore, p53 activity can be induced by oxidative stress [58]; hence, it is reasonable to assume that exposure to BL and the resulting oxidative stress would lead to an upregulation of p53 and, therefore, p53-mediated apoptosis. Since p53 plays an important role when it comes to DNA repair and apoptosis, we assumed that inhibition of p53 would rescue the cells from undergoing apoptosis. By using two p53 inhibitors (pifithrin α and µ), we demonstrated that the induction of caspase 3/7 activity in MCs due to BL exposure could be significantly inhibited (Figure 6B), combined with increased cell viability (Figure 6C). Since pifithrin µ only blocks the mitochondrial binding of p53 to the antiapoptotic BCL-2, thereby reducing the amount of free pro-apoptotic BAX (Figure 5), it can be concluded that apoptosis induced by BL and mediated by p53 is mainly controlled by BAX. Of course, pifithrin µ treatment may also block unknown interactions of p53 with other p53 targets. BL-induced oxidative stress levels could only be significantly reduced by pifithrin α treatment (Figure 6A), evidencing that other (pro-oxidative) p53-dependent genes are probably expressed after pifithrin µ treatment. Under high (oxidative) stress levels, p53 can induce pro-oxidants such as PIG3, NOX1, or POX, further increasing the ROS level [59,60]. Inhibition via pifithrin α could, therefore, prevent the expression of these pro-oxidants, but not pifithrin µ. Hence, enhanced oxidative stress levels, a common aspect of many retinal diseases, can be significantly reduced by p53 inhibition via pifithrin α. The viability of MCs could also be increased by pifithrin α to a level even above the control despite exposure (Figure 6D). Likewise, the significantly reduced cell number 24 h after BL exposure was significantly increased by pifithrin α, while treatment with pifithrin α also resulted in fewer PI-positive and round MCs (Figure 7C,D). Furthermore, treatment with pifithrin α prevented the ATP drop 24 h after BL exposure (Figure 6E). Treatment with pifithrin µ also enhanced cell viability and ATP levels 24 h after BL exposure (Figure 6G,H). Therefore, BL exposure probably leads to p53-mediated expression of pro-oxidative genes, as only pifithrin α treatment was able to significantly reduce oxidative stress levels, and it further proceeds via the mitochondrial pathway since pifithrin µ treatment also decreased caspase 3/7 levels and enhanced cell viability. Taken together, inhibition of p53 by pifithrin α or µ rescues MCs from BL-induced apoptosis, evidencing that BL-dependent apoptosis is probably p53-mediated in MCs. To prevent the loss of MCs in oxidative stress-based retinal diseases, p53 is, therefore, a good therapeutic target.

In contrast to the data generated in MCs, MIO-M1 cells were only slightly affected by BL exposure and enhanced oxidative stress levels (Figure 1B, Figure S1). They demonstrated only a slight increase in caspase 3/7 levels after 6 h (Figure 1B), which were extremely low compared to MCs (Figure 1A). Likewise, cell density and viability returned to control levels 24 h after exposure (Figure 1B). With respect to PL and RL exposure, PL exposure only very slightly enhanced oxidative stress levels and caspase 3/7 activation in MIO-M1 cells (Figure 4A,B). Likewise, no exposure (BL, PL, or RL) led to a reduction in cell number 24 h later (Figure 4D). In accordance with this, cell viability was also only slightly reduced 24 h after BL or PL irradiation. This observation is in strong accordance with the published findings of preserved viability and function in H_2_O_2_-treated MIO-M1 cells [61], suggesting that immortalization of these cells blocks oxidative stress-based apoptosis. Regarding BL-induced apoptosis in MC, this blockade may be caused by a p53 mutation. Nevertheless, enhanced oxidative stress levels (Figure S1B) could result in an impairment of MIO-M1 function, despite the preserved viability, as seen in starved and H_2_O_2_-exposed MIO-M1 cells [56]. 

In summary, BL-induced apoptosis in MCs seems to be p53-dependent, as inhibition of p53 leads to significant improvement and can prevent the cell death of MCs. Therefore, p53 represents a therapeutic target to prevent the death of MCs due to increased oxidative stress levels, thus also preventing the progression of oxidative stress-based diseases such as AMD. Furthermore, it is of great interest to investigate the influence of BL or oxidative stress on other retinal cells with respect to p53 in more detail.

## 4. Materials and Methods

### 4.1. Primary Cells and Cell Lines 

Porcine eyes were obtained from a local abattoir, which euthanized the pigs by electrocution. The eyes were immediately transported at 4 °C to the laboratory to ensure a maximum time of 3 h after death of the animals. The explants were prepared from 6-month-old pigs weighing 100 kg on average. Within 1 h after arrival of the eyes at the lab, retinal explants or primary cells were obtained. As a first step, eyes were cleaned and disinfected in 70% ethanol for 5 min. After two washing steps, they were opened using a scalpel under a laminar flow hood. The cornea, lens, and vitreous humor were removed.

#### 4.1.1. Isolation of Müller Cell-Derived Cultures (MC)

Müller glia cells were isolated by digestion of the porcine retinas with 1% papain (Thermo Fisher Scientific, Karlsruhe, Germany) and DNase I (AppliChem, Darmstadt, Germany) for 15 min at 37 °C with gentle shaking (900 rpm) in a thermo shaker (VWR, Darmstadt, Germany) until homogeneous. After centrifugation (table centrifuge, 5 min) the cell pellet was resuspended in MC culture medium (low-glucose DMEM (Thermo Fisher Scientific, Karlsruhe, Germany) supplemented with 5% fetal bovine serum (FBS, Merck, Darmstadt, Germany) and 1% penicillin/streptomycin (P/S, Gibco, Germany), and the entire cell suspension was transferred into 0.1% gelatin-coated culture vessels (Thermo Fisher Scientific, Karlsruhe, Germany). By replacing the medium until cells were confluent and passaging them into freshly-coated dishes, a pure culture of MCs was obtained after three passages (identified by morphology and immunostaining against GFAP and vimentin) [62]. Cells were cultivated at 5% CO_2_ at 37 °C in an incubator. No passages higher than five were used.

#### 4.1.2. Cultivation of MIO-M1 Cell Line

The human Müller cell line Moorfields/Institute of Ophthalmology–Müller 1 (MIO-M1) was obtained from the UCL Institute of Ophthalmology, London, UK [63]. MIO-M1 cells were cultivated in 5% CO_2_ at 37 °C in an incubator. The MIO-M1 culture medium contained high-glucose DMEM (Thermo Fisher Scientific, Karlsruhe, Germany) supplemented with 5% FBS and 1% P/S.

### 4.2. Light Sources and Exposure Setup

#### Blue, Red, Purple, and White Light Exposure

MCs and MIO-M1 cells were exposed to BL with an intensity of 30 mW/cm^2^ and a peak at 455–465 nm wavelength (3361.1 lx, 5630 SMD Chip, AquaLight lamp Nr. 117377, Mrutzek Meeresaquaristik GmbH, Ritterhude, Germany), applied from below. During the exposure, the temperature was monitored. Furthermore, low-intensity BL exposure of retinal cells (15 mW/cm^2^) was applied from below (peak at 452 nm, LED light strip, C&E Connection E-Commerce (DE) GmbH, Frankfurt, Germany). Purple light (PL) exposure was a combination of blue and red light (peaks at 450/630 nm) with an intensity of 15 mW/cm^2^ (3813.6 lx), applied from below (LED light strip, C&E Connection E-Commerce (DE) GmbH, Frankfurt, Germany). Retinal cells were exposed to WL with an intensity of 30 mW/cm^2^ (66,599 lx, 100,033 K, AquaLight lamp Nr. 117377, with only white light switched on, Mrutzek Meeresaquaristik GmbH, Ritterhude, Germany), applied from below. Red light (RL) exposure (630 nm) with an intensity of 3 mW/cm^2^ was applied from below (LED light strip, C&E Connection E-Commerce (DE) GmbH, Frankfurt, Germany). The irradiation and subsequent cultivation time varied according to cell type and experiment, as indicated in each experiment’s description. Wavelengths were measured using a MAVOSPEC BASE analyzer (Gossen, Nürnberg, Germany).

### 4.3. Methods

#### 4.3.1. Cell Viability, Cell Density, ROS ASSAY, Caspase 3/7 Activity, and ATP Assay

All assays were performed in 96-well plates. A total of 15,000 MIO-M1 cells or 20,000 MCs per well were seeded the day before the experiment (MCs on 0.1% gelatin-coated well plates), resulting in 80–90% confluency. To evaluate cell viability, transparent 96-well plates (Merck, Darmstadt, Germany) and a CellTiter 96^®^ Aqueous One Solution Cell Proliferation Assay (MTS, Promega, Walldorf, Germany) were used according to the manufacturer’s protocol. Absorbance was measured (490/690 nm ratio) using a Tecan Reader (NanoQuant infinite M200) [64]. In this context, the CellTiter Glo 2D Assay (Promega, Walldorf, Germany) was also used to evaluate ATP content, according to the manufacturer’s protocol. Luminescence was measured using a luminometer (Tecan reader SPARK 10M). The results were presented in relative light units (RLUs). To determine cell density, cells were fixed with 4% PFA (paraformaldehyde, Merck, Darmstadt, Germany, 15 min), washed, and incubated with crystal violet solution (Sigma-Aldrich, Taufkirchen, Germany). After 30 min, cells were carefully washed until the water remained clear; then, 1% sodium dodecyl sulfate (SDS, Applichem, Darmstadt, Germany) was added before further incubating for 1 h at RT. Absorbance was then measured using a Tecan Reader (NanoQuant infinite M200) at 595 nm. Promega’s ROS-Glo™ H_2_O_2_ assay and white 96-well plates with a clear bottom (Merck, Darmstadt, Germany) were used to determine the level of hydrogen peroxide in the retinal cell cultures. The assay was performed according to the manufacturer’s instructions (lytic version). Luminescence was measured using a luminometer (Tecan reader SPARK 10M). Activity of caspase 3/7 was determined using a corresponding Caspase Glo Assay from Promega, according to the manufacturer’s protocols [65]. The amount of luminescence was proportional to the amount of caspase activity in the sample. The results were presented in relative light units (RLUs).

#### 4.3.2. Inhibition of p53

p53 was inhibited using pifithrin α (cyclic pifithrin-⍺-*p*-nitro, ab1460 Abcam, Cambdrige, UK) or µ (pifithrin-µ, ab120886, Abcam, Cambridge, UK) at the indicated concentrations (1–10 µM) in a culture medium containing MCs for a certain amount of time. Stock solutions (100 µM) in low-glucose DMEM were aliquoted and frozen at −20 °C. Aliquots were used only once and further diluted in culture medium.

#### 4.3.3. Human Apoptosis Array

To semi-quantitatively investigate apoptotic protein expression, the human apoptosis array C1 (AAH-APO-1-2, HÖLZEL, Köln, Germany) was performed according to the manufacturer’s protocol. Briefly, 2 mg/mL cell lysate was used, which was obtained using the Cell Lysate Buffer + Protease Inhibitor Cocktail of the kit (according to the manufacturer’s instructions). Membranes were incubated with the lysate for 5 h at RT after blocking. Treatment with the primary antibody occurred overnight. After washing steps, incubation with the Biotinylated Antibody Cocktail was performed for 1.5 h at RT. After washing again, the membranes were treated with HRP–streptavidin for 2 h at RT, washed again, and incubated with detection solution; then, the chemiluminescence signal was recorded (2 min, Odyssey Fc Imaging System, LI-COR, Bad Homburg vor der Höhe, Germany). The analysis was performed according to the manufacturer’s instructions, followed by statistical processing with GraphPad Prism 9 (GraphPad software, San Diego, CA, USA).

#### 4.3.4. Annexin V/Propidium Iodide Staining

For annexin V/propidium iodide (PI) staining, 80,000 MCs were seeded in a 24-well plate 24 h before exposing them to BL or WL. After exposure (BL or WL for 1.5 h), MCs were washed with 1× Dulbecco’s phosphate-buffered saline (DBPS, Thermo Fisher Scientific, Karlsruhe, Germany) and stained with annexin V/FITC (Biolegend, San Diego, USA, 5 µM (1:200 in Hanks’ balanced salt solution, HBSS), Thermo Fisher Scientific, Taufkirchen, Germany) and propidium iodide (PI, Sigma-Aldrich, Taufkirchen, Germany, 15 µM (1:100 in HBSS)) for 30 min in the dark. After another washing step, cells were counterstained with Hoechst (Hoechst 3324, 10 mg/mL in H_2_O stock solution, diluted 1:2000 in DPBS, Merck, Darmstadt, Germany) for 5 min, washed, and imaged using a fluorescence microscope with FITC (Annexin V) and AF555 (PI) filter sets [65]. Five pictures per well were generated (one in the middle, one at the top, one to the right, one at the bottom, and one to the left), and colocalization was analyzed using ImageJ. Non-apoptotic cells were defined as DAPI staining without annexin V or PI colocalization. Early apoptotic cells were represented by annexin V/DAPI staining that did not colocalize with PI. Late apoptotic cells or necrotic cells exhibited annexin V/PI colocalization.

#### 4.3.5. Propidium Iodide Staining (Dead Staining)

After BL exposure (and treatment with 1 µM pifithrin α) in a 96-well plate (black, transparent bottom, Falcon, München, Germany), MCs were stained with PI (1:100 in DPBS, 15 µM) and Hoechst (3324, 10 mg/mL in H_2_O stock solution diluted 1:2,000 in DPBS) for 15 min in the dark at RT. After washing with 1× DPBS, cells were imaged in serum-free DMEM using a fluorescence microscope with AF555 (PI) and DAPI (Hoechst) filter sets. Five pictures per well were generated (see previous section). PI-positive cells were counted using the “Analyze particles” function of ImageJ (National Institutes of Health, Bethesda, MD, USA) after adjusting brightness/contrast and threshold (for a converted 16-bit image). Hoechst staining was evaluated analogously (equal to the number of cells).

#### 4.3.6. Quantification of Round MCs

After BL exposure and treatment with 1 µM pifithrin α, bright-field images of MCs were generated (five pictures/well). Round cells were manually counted using ImageJ (Figure 8). Only completely round cells were considered to minimize subjective interpretation. Furthermore, images were evaluated by a blinded investigator. 

#### 4.3.7. Statistical Analysis 

All results were presented as the mean ± SEM. Statistical analysis was performed using GraphPad PRISM 9. Welch’s ANOVA, Kruskal–Wallis, or Welch’s *t*-tests were used for comparison, as indicated in the figure legends. Differences were considered significant at *p* < 0.05. Statistical differences were indicated as follows: * *p* < 0.05, ** *p* < 0.01, *** *p* < 0.001, and **** *p* < 0.0001 compared to the control. In some cases, for better visibility, the control was set to 100%; therefore, bar graphs represent the mean values as percentages, with control values considered as the baseline.

#### 4.3.8. Image Generation

Figures were generated using www.biorender.com (accessed on 12 October 2022).

## 5. Conclusions

Retinal glia cells undergo morphological and functional changes with aging, and these impairments affect the status of all cell types in the retina. Moreover, during AMD progress, MCs begin to migrate through the retina and are activated, likely enhancing inflammation and neuronal cell death. Consequently, damaged and activated MCs can contribute significantly to the progress and development of oxidative stress-based diseases such as AMD. In this context, we were able to prove that BL enhances oxidative stress levels and induces p53-mediated apoptosis. In the case of an insufficient clearance of apoptotic MCs in vivo, this could also lead to secondary necrosis and, thus, further inflammation in the retina. During AMD, MCs could even reach RPE cells, which are not typically in direct contact. Therefore, damaged MCs could also impair RPE function and further enhance AMD progression. Importantly, BL-induced apoptosis can be reduced by p53 inhibition, highlighting a promising therapeutic option. Using ex vivo organ cultures, the effect of BL-damaged MCs could be studied in a more complex context. In particular, the influence on other retinal cell types is an exciting topic of research, as is the question of whether retinal cell damage by increased oxidative stress can be mediated by inhibiting p53.

## Supplementary Materials

The supporting information can be downloaded at: https://www.mdpi.com/article/10.3390/ijms232314540/s1.

## References

[1] Villette T. (2013). Blue Light Hazard: New Knowledge, New Approaches to Maintaining Ocular Health.

[2] Nakamura S., Chichibu S.F. (2000). Introduction to Nitride Semiconductor Blue Lasers and Light Emitting Diodes.

[3] O’Hagan J.B., Khazova M., A Price L.L. (2016). Low-energy light bulbs, computers, tablets and the blue light hazard. Eye.

[4] Peng M., Tsai C., Chien C., Hsiao J.C., Huang S., Lee C., Lin H., Wen Y., Tseng K. (2012). The Influence of Low-powered Family LED Lighting on Eyes in Mice Experimental Model. Life Sci. J..

[5] Pardhan S., Sapkota R.P. (2016). Eye complications of exposure to blue-violet light. Points Vue Int. Rev. Ophthalmic Opt..

[6] Dunaief J.L., Dentchev T., Ying G.S., Milam A.H. (2002). The role of apoptosis in age-related macular degeneration. Arch. Ophthalmol..

[7] Turner P.L., Mainster M.A. (2008). Circadian photoreception: Ageing and the eye’s important role in systemic health. Ophthalmology.

[8] Behar-Cohen F., Martinsons C., Viénot F., Zissis G., Barlier-Salsi A., Cesarini J.P., Enouf O., Garcia M., Picaud S., Attia D. (2011). Light-emitting diodes (LED) for domestic lighting: Any risks for the eye?. Prog. Retin. Eye Res..

[9] Czepita D., Mojsa A., Ustianowska M., Czepita M., Lachowicz E. (2010). Reading, writing, working on a computer or watching television, and myopia. Klin Oczna.

[10] Lou L., Arumugam B., Hung L.F., Beach K.M., She Z., Smith E.L., Ostrin L.A. (2021). Effects of narrowband light rearing on activity and the pupil in infant rhesus monkeys. Investig. Ophthalmol. Vis. Sci..

[11] Jaadane I., Villalpando Rodriguez G., Boulenguez P. (2020). Retinal phototoxicity and the evaluation of the blue light hazard of a new solid-state lighting technology. Sci. Rep..

[12] Moon J., Yun J., Yoon Y.D., Park S.I., Seo Y.J., Park W.S., Chu H.Y., Park K.H., Lee M.Y., Lee C.W. (2017). Blue Light Effect on Retinal Pigment Epithelial Cells by Display Devices. Integr. Biol..

[13] Hunter J.J., Morgan M.J., Merigan W.H., Sliney D.H., Sparrow J.R., Williams D.R. (2012). The susceptibility of the retina to photochemical damage from visible light. Prog. Retin. Eye Res..

[14] Margrain T.H., Boulton M., Marshall J., Sliney D.H. (2004). Do blue light filters confer protection against age-related macular degeneration?. Prog. Retin. Eye Res..

[15] Lu L., Oveson B.C., Jo Y.J., Lauer T.W., Usui S., Komeima K., Xie B., Campochiaro P.A. (2009). Increased expression of glutathione peroxidase 4 strongly protects retina from oxidative damage. Antioxid. Redox Signal..

[16] Algvere P.V., Marshall J., Seregard S. (2006). Age-related maculopathy and the impact of blue light hazard. Acta Ophthalmol. Scand..

[17] Sachdeva M.M., Cano M., Handa J.T. (2014). Nrf2 signaling is impaired in the aging RPE given an oxidative insult. Exp. Eye Res..

[18] Cai J., Nelson K.C., Wu M., Sternberg P., Jones D.P. (2000). Oxidative damage and protection of the RPE. Prog. Retin. Eye Res..

[19] Arnault E., Barrau C., Nanteau C., Gondouin P., Bigot K., Viénot F., Gutman E., Fontaine V., Villette T., Cohen-Tannoudji D. (2013). Phototoxic action spectrum on a retinal pigment epithelium model of age-related macular degeneration exposed to sunlight normalized conditions. PLoS ONE.

[20] Jarrett S.G., Boulton M.E. (2012). Consequences of oxidative stress in age-related macular degeneration. Mol. Aspects Med..

[21] Masuda T., Boulton S.M., Hara H. (2017). Retinal Diseases Associated with Oxidative Stress and the Effects of a Free Radical Scavenger (Edaravone). Oxid. Med. Cell. Longev..

[22] Sasaki M., Yuki K., Kurihara T., Miyake S., Noda K., Kobayashi S., Ishida S., Tsubota K., Ozawa Y. (2012). Biological role of lutein in the light-induced retinal degeneration. J. Nutr. Biochem..

[23] Ozawa Y. (2020). Oxidative stress in the light-exposed retina and its implication in age-related macular degeneration. Redox Biol..

[24] Chen Y., Sawada O., Kohno H., Le Y.Z., Subauste C., Maeda T., Maeda A. (2013). Autophagy protects the retina from light-induced degeneration. J. Biol. Chem..

[25] Ban N., Ozawa Y., Osada H., Lin J.B., Toda E., Watanabe M., Yuki K., Kubota S., Apte R.S., Tsubota K. (2017). Neuroprotective role of retinal SIRT3 against acute photo-stress. NPJ Aging Mech. Dis..

[26] Spector A. (1995). Oxidative stress-induced cataract: Mechanism of action. FASEB J..

[27] Imamura Y., Noda S., Hashizume K., Shinoda K., Yamaguchi M., Uchiyama S., Shimizu T., Mizushima Y., Shirasawa T., Tsubota K. (2006). Drusen, choroidal neovascularization, and retinal pigment epithelium dysfunction in SOD1-deficient mice: A model of age-related macular degeneration. Proc. Natl. Acad. Sci. USA.

[28] Gritz D.C., Montes C., Atalla L.R., Wu G.S., Sevanian A., Rao N.A. (1991). Histochemical localization of superoxide production in experimental autoimmune uveitis. Curr. Eye Res..

[29] Niesman M.R., Johnson K.A., Penn J.S. (1997). Therapeutic effect of liposomal superoxide dismutase in an animal model of retinopathy of prematurity. Neurochem. Res..

[30] Alio J.L., Ayala M.J., Mulet M.E., Artola A., Ruiz J.M., Bellot J. (1995). Antioxidant therapy in the treatment of experimental acute corneal inflammation. Ophthalmic Res..

[31] Ham W.T., Mueller H.A., Ruffolo J.J., Millen J.E., Cleary S.F., Guerry R.K., Guerry D. (1984). Basic mechanisms underlying the production of photochemical lesions in the mammalian retina. Curr. Eye Res..

[32] Iandiev I., Wurm A., Hollborn M., Wiedemann P., Grimm C., Remé C.E., Reichenbach A., Pannicke T., Bringmann A. (2008). Müller Cell Response to Blue Light Injury of the Rat Retina. Investig. Ophthalmol. Vis. Sci..

[33] Cuenca N., Fernandez-Sanchez L., Campello L., Maneu V., De la Villa P., Lax P., Pinilla I. (2014). Cellular responses following retinal injuries and therapeutic approaches for neurodegenerative diseases. Prog. Retin. Eye Res..

[34] Subirada P.V., Paz M.C., Ridano M.E., Lorenc V.E., Vaglienti M.V., Barcelona P.F., Luna J.D., Sánchez M.C. (2018). A Journey Into the Retina: Müller Glia Commanding Survival and Death. Eur. J. Neurosci..

[35] Bora N.S., Matta B., Lyzogubov V.V., Bora P.S. (2015). Relationship between the complement system, risk factors and prediction models in age-related macular degeneration. Mol. Immunol..

[36] Edwards M.M., McLeod D.S., Bhutto I.A., Villalonga M.B., Seddon J.M., Lutty G.A. (2016). Idiopathic preretinal glia in aging and age-related macular degeneration. Exp Eye Res..

[37] Franze K., Grosche J., Skatchkov S.N., Schinkinger S., Foja C., Schild D., Uckermann O., Travis K., Reichenbach A., Guck J. (2007). Muller cells are living optical fibers in the vertebrate retina. Proc. Natl. Acad. Sci. USA.

[38] Ardeljan D., Chan C.C. (2013). Aging is not a disease: Distinguishing age-related macular degeneration from aging. Prog. Retin. Eye Res..

[39] Beatty S., Koh H.H., Henson D., Boulton M. (2000). The role of oxidative stress in the pathogenesis of age-related macular degeneration. Surv. Ophthalmol..

[40] Sun Q., Kim H.E., Cho H., Shi S., Kim B., Kim O. (2018). Red light-emitting diode irradiation regulates oxidative stress and inflammation through SPHK1/NF-κB activation in human keratinocytes. J. Photochem. Photobiol. B Biol..

[41] Zhang J., Yue X., Luo H., Jiang W., Mei Y., Ai L., Gao G., Wu Y., Yang H., An J. (2019). Illumination with 630 nm Red Light Reduces Oxidative Stress and Restores Memory by Photo-Activating Catalase and Formaldehyde Dehydrogenase in SAMP8 Mice. Antioxid. Redox Signal..

[42] Núñez-Álvarez C., Suárez-Barrio C., del Olmo Aguado S., Osborne N.N. (2018). Blue light negatively affects the survival of ARPE19 cells through an action on their mitochondria and blunted by red light. Acta Ophthalmol. Scand. Found..

[43] Godley B.F., Shamsi F.A., Liang F.Q., Jarrett S.G., Davies S., Boulton M. (2005). Blue light induces mitochondrial DNA damage and free radical production in epithelial cells. J. Biol. Chem..

[44] Mueller-Buehl A.M., Doepper H., Grauthoff S., Kiebler T., Peters L., Hurst J., Kuehn S., Bartz-Schmidt K.U., Dick H.B., Joachim S.C. (2020). Oxidative stress-induced retinal damage is prevented by mild hypothermia in an ex vivo model of cultivated porcine retinas. Clin. Exp. Ophthalmol..

[45] Cervellati F., Cervellati C., Romani A., Cremonini E., Sticozzi C., Belmonte G., Pessina F., Valacchi G. (2014). Hypoxia induces cell damage via oxidative stress in retinal epithelial cells. Free Radic. Res..

[46] Kuehn S., Hurst J., Rensinghoff F., Tsai T., Grauthoff S., Satgunarajah Y., Dick H.B., Schnichels S., Joachim S.C. (2017). Degenerative effects of cobalt-chloride treatment on neurons and microglia in a porcine retina organ culture model. Exp. Eye Res..

[47] Sarna T. (1992). Properties and function of the ocular melanin—A photobiophysical view. J. Photochem. Photobiol..

[48] Boulton M., Różanowska M., Różanowski B. (2001). Retinal photodamage. J. Photochem. Photobiol..

[49] Hollborn M., Ulbricht E., Rillich K., Dukic-Stefanovic S., Wurm A., Wagner L., Reichenbach A., Wiedemann P., Limb G.A., Bringmann A. (2011). The human Müller cell line MIO-M1 expresses opsins. Mol. Vis..

[50] Rios M.N., Marchese N.A., Guido M.E. (2019). Expression of Non-visual Opsins Opn3 and Opn5 in the Developing Inner Retinal Cells of Birds. Light-Responses in Müller Glial Cells. Front. Cell. Neurosci..

[51] Kuse Y., Ogawa K., Tsuruma K., Shimazawa M., Hara H. (2014). Damage of photoreceptor-derived cells in culture induced by light emitting diode-derived blue light. Sci. Rep..

[52] Mellerio J. (1994). Light effects on the retina. Princ. Pract. Ophthalmol. Basic Sci..

[53] García-Silva M.T., Ribes A., Campos Y., Garavaglia B., Arenas J. (1997). Syndrome of encephalopathy, petechiae, and ethylmalonic aciduria. Pediatr. Neurol..

[54] Hockberger P.E., Skimina T.A., Centonze V.E., Lavin C., Chu S., Dadras S., Reddy J.K., White J.G. (1999). Activation of flavin-containing oxidases underlies light-induced production of H2O2 in mammalian cells. Proc. Natl. Acad. Sci. USA.

[55] Helm K., Beyreis M., Mayr C., Ritter M., Jakab M., Kiesslich T., Plaetzer K. (2017). Cell Death Discrimination and Screening Method by Simple and Cost-Effective Viability Analysis. Cell. Physiol. Biochem..

[56] Peters S., Griebsch M., Klemm M., Haueisen J., Hammer M. (2017). Hydrogen peroxide modulates energy metabolism and oxidative stress in cultures of permanent human Müller cells MIO-M1. J. Biophotonics.

[57] Jeon C.J., Strettoi E., Masland R.H. (1998). The major cell populations of the mouse retina. J. Neurosci..

[58] Yamamoto H., Ozaki T., Nakanishi M., Kikuchi H., Yoshida K., Horie H., Kuwano H., Nakagawara A. (2007). Oxidative stress induces p53-dependent apoptosis in hepatoblastoma cell through its nuclear translocation. Genes Cells.

[59] Liu D., Xu Y. (2011). p53, oxidative stress, and aging. Antioxid. Redox Signal..

[60] Simabuco F.M., Morale M.G., Pavan I.C., Morelli A.P., Silva F.R., Tamura R.E. (2018). p53 and metabolism: From mechanism to therapeutics. Oncotarget.

[61] Toft-Kehler A.K., Gurubaran I.S., Desler C., Rasmussen L.J., Skytt D.M., Kolko M. (2016). Oxidative Stress-Induced Dysfunction of Müller Cells During Starvation. Invest. Ophthalmol. Vis. Sci..

[62] Pereiro X., Ruzafa N., Acera A., Urcola A., Vecino E. (2020). Optimization of a Method to Isolate and Culture Adult Porcine, Rats and Mice Müller Glia in Order to Study Retinal Diseases. Front. Cell. Neurosci..

[63] Limb G.A., Salt T.E., Munro P.M., Moss S.E., Khaw P.T. (2002). In vitro characterization of a spontaneously immortalized human Müller cell line (MIO-M1). Investig. Ophthalmol. Vis. Sci..

[64] Schnichels S., Hagemann U., Januschowski K., Hofmann J., Bartz-Schmidt K.U., Szurman P., Spitzer M.S., Aisenbrey S. (2013). Comparative toxicity and proliferation testing of aflibercept, bevacizumab and ranibizumab on different ocular cells. Br. J. Ophthalmol..

[65] Schnichels S., Schultheiß M., Hofmann J., Szurman P., Bartz-Schmidt K.U., Spitzer M.S. (2012). Trichostatin A induces cell death at the concentration recommended to differentiate the RGC-5 cell line. Neurochem. Int..

