# Supplementary material for: Out of the Shadow: Blue Light Exposure Induces Apoptosis in Müller Cells
Source: Int J Mol Sci. 2022 Nov 22;23(23):14540. doi: 10.3390/ijms232314540 (PMC9739907; doi:10.3390/ijms232314540)
Supplement: Supplementary file 1 [file ijms-23-14540-s001.zip › ijms-2000891-supplementary (1).pdf]

International Journal of *Molecular Sciences*

MDPI

Supplementary Materials

Out of the shadow: Blue light exposure induces p53-dependent apoptosis in Müller cells

Agnes Fietz [1],José Hurst [1]*and Sven Schnichels [1]

[1] Center for Ophthalmology Tübingen, University Eye Hospital Tübingen, 72076 Tübingen, Germany
* Correspondence: jose.hurst@med.uni-tuebingen.de; Tel.: +4970712987883

Table S1. Original data for Figure 1: controls and blue light-exposed cells.

Cell type	Time point (h)	ROS	Caspase 3/7	Viability	Density
MCs	1		50596		
	2	44657	99022		
	4	44694	65507		
	6	44081	89078	0,6935	1,211
	12	49120	95779	0,8573	0,6647
	18		56630	0,9056	0,8931
	24	95923	321121	0,8194	0,7697
MIO-M1	6		51313	0,79156	0,90404
	12		106034	0,49139	0,66764
	24		155200	0,43688	0,76965

Cell type	Time point (h)	ROS	Caspase 3/7	Viability	Density
MCs	1		45436		
	2	108814	51359		
	4	180752	120817		
	6	494176	372101	0,9056	1,293
	12	257184	687723	0,7746	0,5687
	18		343189	0,8106	0,7900
	24	48931	492049	0,4804	0,4487
MIO-M1	6		76186	0,78511	0,85155
	12		77495	0,42195001	0,56582
	24		195092	0,33501	0,71847001

The original data of Figure 2 are given as average values. The first table contains the controls, while the second table contains the blue light-exposed cells.

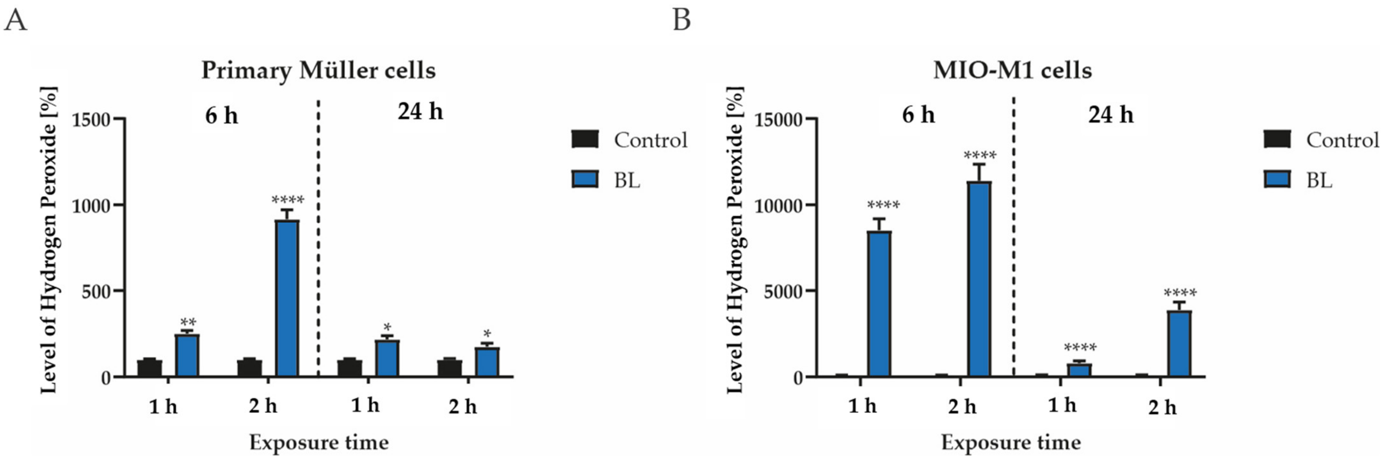

Figure S1. Oxidative stress can be induced in porcine Müller cell-derived cells, as well as in corresponding cell line MIO-M1, by blue light exposure. (**A**) Müller cell-derived cultures (MCs) and (**B**) human MIO-M1 cells were exposed to blue light (BL, 30 mW/cm²) for 1 or 2 h and cultivated for 6 or 24 h. The level of hydrogen peroxide was measured. After both 6 and 24 h, a significant and dose-dependent increase was detected in MC and MIO-M1 cells; $n = 10$. (Bar graphs represent the mean values and SEM. The control was set to 100%. Statistical differences are indicated as follows: * $p < 0.05$, ** $p < 0.01$, *** $p < 0.001$, and **** $p < 0.0001$ compared to control, according to Welch's one-way ANOVA.

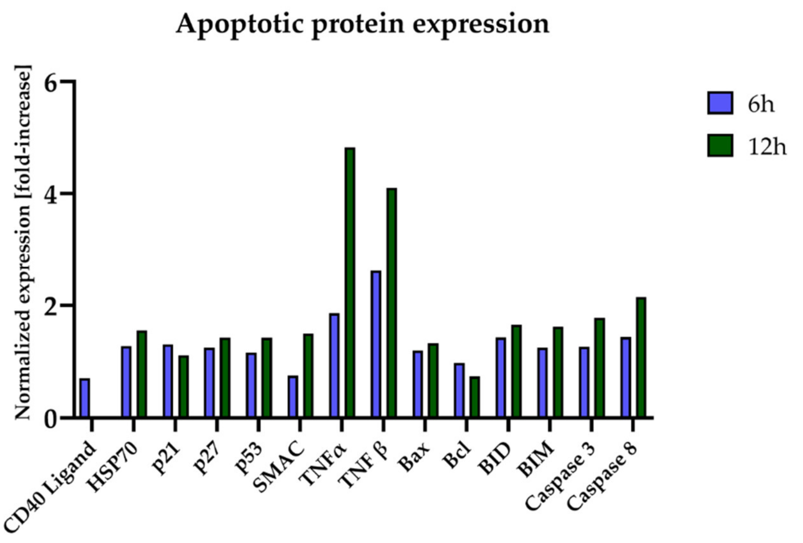

Figure S2. Pro-apoptotic protein expression was increased due to blue light exposure in porcine Müller cell-derived cells. Müller cell derived cells (MCs) were exposed to blue light (BL, 30 mW/cm²) and further cultivated for 6 or 12 h. Cell lysates were collected, and the human apoptosis array was performed with 2 mg/mL lysate/condition. Values were normalized to the control and represent fold-changes. An overall increase in pro-apoptotic protein expression was observed with an increase over time (6 to 12 h of cultivation). Five different samples were pooled for each condition ($n = 5$); the assay was performed once. Duplicates for each protein were used; background values were subtracted from mean values and normalized to a positive control for each membrane/condition. Normalized values were compared to those from the unexposed controls, and the fold-increase is shown.

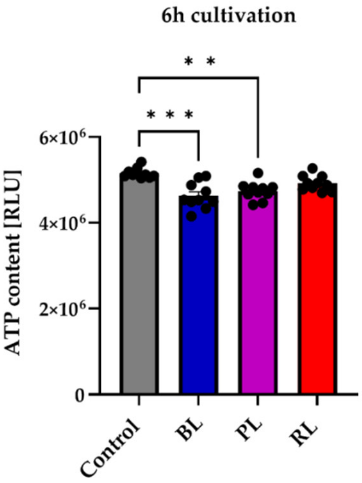

Figure S3. ATP content was only slightly decreased due to blue- or purple-light exposure, but not red light in porcine Müller cell-derived cells. Müller cell-derived cells (MCs) were exposed to blue (BL), purple (PL), or red light (RL) for 1.5 h and further cultivated for 6 h. Evaluation of ATP content revealed a slight decrease due to BL or PL exposure, but not due to RL. Bar graphs represent the mean values and SEM. Statistical differences are indicated as follows: * $p < 0.05$, ** $p < 0.01$, *** $p < 0.001$, and **** $p < 0.0001$ compared to control, according to Welch's one-way ANOVA.
